# White Matter Atrophy in Patients with Mesial Temporal Lobe Epilepsy: Voxel-Based Morphometry Analysis of T1- and T2-Weighted MR Images

**DOI:** 10.1155/2012/481378

**Published:** 2012-10-24

**Authors:** Barbara Braga, Clarissa L. Yasuda, Fernando Cendes

**Affiliations:** Laboratory of Neuroimaging, Department of Neurology, University of Campinas (UNICAMP), Campinas, SP, Brazil

## Abstract

*Introduction*. Mesial temporal lobe epilepsy (MTLE) associated with hippocampal sclerosis is highly refractory to clinical treatment. MRI voxel-based morphometry (VBM) of T1-weighted images has revealed a widespread pattern of gray matter (GM) and white matter (WM) atrophy in MTLE. Few studies have investigated the role of T2-weighted images in revealing WM atrophy using VBM. *Objectives*. To compare the results of WM atrophy between T1- and T2-weighted images through VBM. *Methods*. We selected 28 patients with left and 27 with right MTLE and 60 normal controls. We analyzed T1- and T2- weighted images with SPM8, using VBM/DARTEL algorithm to extract maps of GM and WM. The second level of SPM was used to investigate areas of WM atrophy among groups. *Results*. Both acquisitions showed bilateral widespread WM atrophy. T1-weighted images showed higher sensibility to detect areas of WM atrophy in both groups of MTLE. T2-weighted images also showed areas of WM atrophy in a more restricted pattern, but still bilateral and with a large area of superposition with T1-weighted images. *Conclusions*. In MTLE, T1-weighted images are more sensitive to detect subtle WM abnormalities using VBM, compared to T2 images, although both present a good superposition of statistical maps.

## 1. Introduction

The mesial temporal lobe epilepsy (MTLE) is highly refractory to pharmacological treatment [1], and it is the main group of epilepsy referred to the tertiary care hospitals for surgical treatment [2]. MTLE shows a good surgical prognosis, with satisfactory seizures control in 60–80% of the patients [3].

In approximately 65% of cases, the MTLE is associated to hippocampal atrophy and other MRI signs of hippocampal sclerosis [4], and the remaining cases are related to other etiologies, like tumors, vascular damage, and cortical malformations [5]. We can also find the so-called dual pathology, in which hippocampal sclerosis is found combined with other affections of central nervous system [6], such as focal cortical dysplasia, vascular malformations, and tumors.

The most accepted hypothesis defends that histopathological abnormalities in hippocampal sclerosis are a multifactorial phenomenon, resulting from interaction between genetic factors [7], early cerebral injury [8], and progressive neuronal loss caused by repetitive seizures [9].

There are patients with MTLE, with or without clear MRI signs of hippocampal sclerosis, that respond well to antiepileptic drugs (AEDs) and are sometimes referred to as benign MTLE [10]. Recent published data [11] show that patients with benign MTLE also have gray matter reductions in regions outside the hippocampus, mainly cortical thinning in sensorimotor areas.

The neuronal atrophy in patients with MLTE is not restricted to hippocampus or ipsilateral temporal lobe; actually, it has been associated to both white matter (WM) and grey matter (GM) atrophy encompassing bilateral extratemporal areas [8] as well as subcortical structures, including thalami [12]. Although the pathophysiology of WM atrophy in MTLE is not yet fully understood, some studies have analyzed the relationship between WM damage and the cognitive dysfunction exhibited by some patients [13]. Among the potential mechanisms involved in WM atrophy, some authors analyzed the neuronal heterotopia [14], microdysgenesis [15], and dysfunctions in the myelination process [16]. We believe that these widespread extratemporal structural abnormalities are probably secondary to both repetitive seizures and other insults such as trauma and infections.

The VBM (voxel-based morphometry) method has been widely used in neuroimaging studies, not only in epilepsy, with the aim of identifying atrophy or excess or encephalic tissues in several brain areas. Our group has applied this technique to investigate brain atrophy in other diseases such as Alzheimer [17], Machado-Joseph's disease [18] and Friedreich's ataxia [19]. In MTLE, most of the studies prioritize the detection of abnormalities of GM, usually revealing areas of atrophy within hippocampus, amygdale, parahippocampal gyrus, fusiform gyrus, and superior temporal gyrus [20]. In extratemporal areas, GM atrophy occurs mainly in basal ganglia, insula, thalamus, cerebellum, frontal, parietal, and occipital cortex [21–23]. WM atrophy in MTLE has been described with VBM studies, in a widespread pattern, encompassing temporal, frontal and occipital lobes, besides the corpus callosum [24, 25].

Previous studies have already demonstrated the pattern of WM atrophy in patients with MTLE using T1-weighted scans [21], but published data indicates that T1 images are not ideal for morphometric analysis of WM as the intensity of signal in T1 is not well correlated with WM integrity [26]. In diseases such as multiple sclerosis, the intensity of WM signal in T1 may be normal, even when there is evident tissue damage [27].

In this work, we aimed to compare the findings of white matter atrophy with VBM analysis between T1- and T2- weighted scans, hypothesizing that T2 acquisition would allow better assessment of the WM integrity.

## 2. Materials and Methods

### 2.1. Patients' Selection

We selected a group of 55 patients (27 women, 42.86 ± 12.70 years old), according to the following inclusion criteria: age older than 12 years old with clinical and electroencephalographic MTLE diagnosis, according to ILAE criteria. Medical history and seizures description were consistent with unilateral MTLE syndrome and verified by review of medical records. The diagnosis of hippocampal atrophy and other signs of hippocampal sclerosis were carried out by visual analysis of MRIs acquired with an epilepsy protocol with thin coronal cuts. The criteria for MRI signs of hippocampal sclerosis included identification of volume reduction and loss of internal structure of hippocampus, with or without hyperintense T2 signal [26]. We only included in this study patients with unilateral MRI signs of hippocampal sclerosis, in order to make the sample more homogeneous. Patients with bilateral hippocampal atrophy or patients with MTLE with normal MRI or other types of lesions were not included.

The mean age of seizure onset was 14.1 years old, and the most common subtype of seizure was complex partial seizure (CPS). This series includes 8 patients, who were seizure-free for more than one year before MRI acquisition and the remaining patients had seizures refractory to AEDs.

We included 60 controls (31 women, 40.95 ± 12.16 years old) without neurological or psychiatric symptoms and normal MRI.

### 2.2. Subgroups according to the Side of Hippocampal Atrophy according to Visual Analyses of MRI

The patients and controls were separated into 3 groups, according to the side of lesions:

- patients with left atrophy (28 patients),
- patients with right atrophy (27 patients),
- controls (60).

### 2.3. Image Acquisition

The MRIs were acquired in a 3T scanner (Philips Achieva), in coronal, sagittal, and axial planes, in addition to 3D acquisitions, that allowed the reconstruction of images in any plane. The coronal images for visual analyses were obtained perpendicular to long axis of hippocampus defined on the sagittal images with thickness of 3 mm, for optimal analysis of this structure, and included T1 inversion recovery, T2-weighted multieco, and FLAIR (fluid-attenuated inversion recovery) images.

All patients and controls had T1-weighted and T2-weighted 3D MRIs for VBM analyses. The T1-weighted 3-dimensional gradient echo with 1 mm isotropic voxels, was acquired in the sagittal plane (flip angle, 8°; TR, 7.1; TE, 3.2; matrix, 240 × 240; FOV, 240 × 240 cm). The T2-weighted 3-dimension fast spin echo with 1.5 mm isotropic voxels was acquired in the sagittal plane (flip angle, 90°; TR, 1800; TE, 342; matrix, 140 × 139; FOV, 210 × 210).

### 2.4. Image Preprocessing for VBM Analysis

The images were obtained from records of MRI sector of the Neuroimaging Laboratory, UNICAMP, during 2009 and 2010. We used MRIcro (http://www.mricro.com/) to transform the original format to ANALYSE and mark the anterior commissure for the normalization process.

### 2.5. Voxel-Based Morphometry

The standard VBM method can be summarized by the following sequence of steps: spatial normalization, segmentation, modulation, and smoothing. After smoothing, each voxel of the image is a locally weighted average of GM or WM density from a region of surrounding voxels, and statistical analyses can be performed [21, 28].

After the preprocessing, we used SPM8 with DARTEL [29] (http://www.fil.ion.ucl.ac.uk/spm/software/spm8/) on MATLAB 7.7 (The Math-Works, Inc., Natick, MA) to proceed with the extraction of GM and WM maps, and registering them to a standard space (MNI space) in order to perform group comparisons [30].

After segmentation, the images were modulated (the automatic multiplication of voxels by the deformity parameters that they were submitted during the normalization process) to compensate the deformity that occurs in normalization, preserving the WM and GM volumes considered in this analysis.

The statistical analysis was performed with SPM8 using full factorial model, with significance of *P* < 0.05, uncorrected for multiple comparisons. Contrasts were built to investigate differences between each group side (right or left) and controls, for T1 and T2 scans. The location of neuroanatomical structures corresponding to the statistically significant areas was performed with xjview (http://www.alivelearn.net/xjview8/).

## 3. Results

The clinical characteristics of patients are summarized in Table 1.

### 3.1. Left MTLE Group

**Figure 1:**
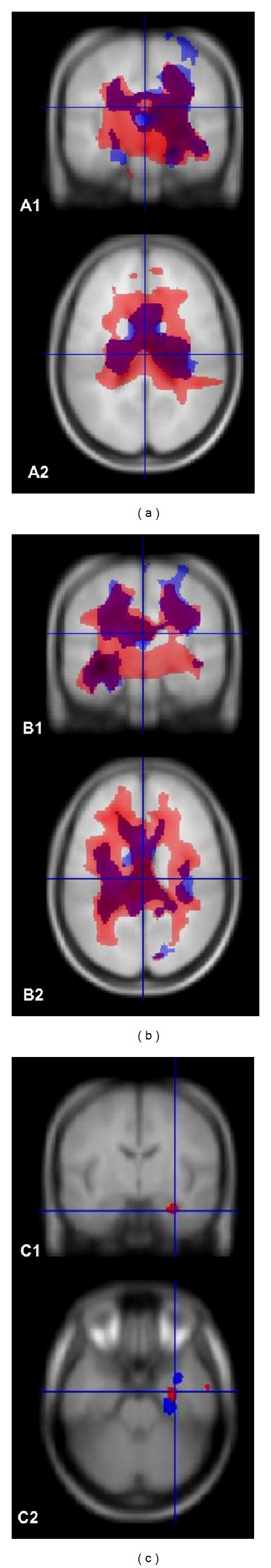
Right side represents left side of the head. (a) A1 and A2: left MTLE; these images show the pattern of atrophy in coronal and axial slices, respectively, in a superposition of T1 and T2 acquisitions. The area colored in red represents areas of WM atrophy in T1-weighted images, and the area in blue represents WM atrophy in T2-weighted images. The intersection T1 and T2 is shown in purple. The figures show a bilateral pattern of WM atrophy, detected by both acquisitions, and a more widespread area in T1-weighted images, despite a considerable area of superposition. (b) B1 and B2: right MTLE; these images show the pattern of atrophy in coronal and axial slices, respectively, in a superposition of T1 and T2 acquisitions. The area colored in red represents WM atrophy in T1-weighted images, and the area in blue represents WM atrophy in T2-weighted images. The intersection T1 and T2 is shown in purple. The figures show a bilateral pattern of WM atrophy, detected by both acquisitions, and a more widespread area of WM atrophy in T1-weighted images, despite of a considerable area of superposition. (c) C1 and C2: left minus right MTLE groups: we made a subtraction between left and right MTLE patients. This subtraction evidentiates a pronounced WM atrophy in the ipsilateral (left) mesial area.

The results are shown in Figures 1(A1) and 1(A2) and Tables 2 and 3.

#### 3.1.1. T1-Weighted Scans

VBM from images in T1 showed a widespread pattern of atrophy, bilateral and not only restricted to mesial area, demonstrating high sensibility to detect WM atrophy. Among the structures identified, we found hippocampus ipsilateral, parahippocampal gyrus, fusiform gyrus, and amygdale. Volumetric decreases were also detected in hippocampus and parahippocampal gyrus, contralaterally. Besides temporal mesial structures, signal changes were also found in thalamus area, caudate, corpus callosum, parietal lobe, insula, lingual gyrus, and anterior portion of cerebellum.

#### 3.1.2. T2-Weighted Scans

VBM from T2-weighted images also showed a bilateral pattern of atrophy. The areas of atrophy were not restricted to mesial temporal area. Among the areas identified with atrophy, we found hippocampus, parahippocampal gyrus, cerebellum, thalamus, corpus callosum, insula, uncus, fusiform gyrus, basal ganglia, and areas in occipital and parietal lobes.

#### 3.1.3. Superposition of T1 and T2 Maps

We found a considerable area of superposition, advising that both acquisitions were able to detect atrophy of WM. These areas were bilateral and not only restricted to temporal region. T1-weighted MRI showed higher sensibility to detect atrophy, revealing a more diffuse pattern, whereas T2-weighted pattern was more restricted to identify areas of WM atrophy.

### 3.2. Right MTLE Group

The results are shown in Figures 1 (B1) and 1(B2) and Tables 4 and 5.

#### 3.2.1. T1-Weighted Scans

We observed a widespread pattern of atrophy, bilateral and not only restricted to mesial area. Among the structures identified, we found hippocampus, parahippocampal gyrus, fusiform gyrus and amygdale, cingulated gyrus, thalamus area, caudate, corpus callosum, parietal lobe, insula, and cerebellum.

#### 3.2.2. T2-Weighted Scans

We identified a bilateral pattern of atrophy. The areas of atrophy were not restricted to mesial temporal area. Among the areas identified with atrophy, we identified cerebellum, corpus callosum, cingulate gyrus, precentral gyrus, thalamus, parahippocampal gyrus, fusiform gyrus, and occipital lobe.

#### 3.2.3. Superposition of T1 and T2 Maps

A considerable area of superposition was identified, suggesting that both acquisitions were able to detect atrophy of WM. These areas were bilateral and not only restricted to temporal region. T1-weighted MRI showed higher sensibility to detect atrophy with a more diffuse pattern, while T2-weighted maps showed more restricted areas of WM atrophy.

### 3.3. Comparison between Right and Left MTLE

Although visual inspection of Figure 1 suggests a more widespread pattern of WM atrophy in the right MTLE group, the statistical difference between right and left MTLE groups pointed exclusively for more intense atrophy of left MTLE group. In Figures 1 (C1) and 1(C2), we showed that left MTLE group presented intense atrophy in the left temporal lobe, when compared exclusively with right MTLE group. On contrary, we did not identify similar results in the right side from the reverse comparison (i.e., SPM 10 contrast set to search for areas of more atrophy in the right MTLE compared to left MTLE group); therefore, our results suggest more intense bilateral WM damage in mesial temporal lobes of left MTLE group.

## 4. Discussion

In our study, we confirmed the presence of WM atrophy in patients with MTLE, not restricted to mesial temporal lobe structures. The volumetric decrease in extratemporal areas, according to Bonilha et al. [30], would be associated to the hippocampus deafferentation, with loss of connection with other areas, in different lobes of the brain. The WM atrophy in the contralateral hemisphere was identified in all groups, in agreement with Keller's findings, suggesting a wider atrophy of cerebral parenchyma [31].

Some previous studies demonstrated association between the extension and the pattern of WM atrophy with cognitive deficits in long-term epilepsy [13]. The pathophysiology of WM atrophy in MTLE is not well elucidated. Mitchell et al. [16] suggest that the persistence of immature myelin would be the cause of a posterior WM atrophy, in patients genetically predisposed or that were exposed to early neuronal injuries. Other studies suggest that primary cortical malformations would have a role in seizures propagation, with secondary effect in neuronal loss [15].

All the tested groups exhibited a similar pattern of volumetric decrease in mesial temporal lobe and within the thalamus, supporting the findings shown by Rosenberg et al. [32] that consider the essential role of thalamus in epileptic seizures propagation.

The hippocampus, amygdale, caudate, and the thalamus were structures with high statistical differences in the WM volume even though these are composed basically by grey matter [33]. This may be explained by the fact that the small proportion of WM within these structures is consistently altered in MTLE patients; therefore, the statistical difference here does not necessarily indicate the degree of changes but its reproducibility among patients.

In both groups of patients (left and right MTLE), the T1-weighted images showed a wider pattern of atrophy than T2-weighted images. T2-weighted images showed also bilateral atrophy, not only restricted to mesial temporal areas, but in a less extensive pattern. Nevertheless, the superposition of T1 and T2 statistical maps showed a considerable and satisfactory area of concordance, that allows the use of both acquisitions with the aim of identify areas of WM atrophy in MTLE.

In general, T1-weighted images are used for anatomic analysis of brain. This sequence elicits the most signal from WM and bone marrow, due to their short T1 values, and an intermediate amount of signal from GM, while a very little signal is recovered from cerebrospinal fluid. By contrast, with T2-weighted images, there is a maximal signal in fluid-filled regions, which is important for many clinical diagnoses of disease processes. High-resolution T2-weighted images are also used as anatomical references in functional MRI studies, isolated or with T1-weighted images. In T2-weighted images, the brightest image is in fluid-filled regions as the ventricles, medium brightness in GM, and darkest within WM [34]. Myelinated WM structures, in normal brain, have a higher signal than GM structures in T1-weighted images and lower signal in T2-weighted images. When demyelination occurs, the contrast is reversed, and WM has lower signal than GM in T1 and a higher signal in T2. With myelin deposition, there is an earlier and more pronounced T1 shortening than T2 shortening. We can first observe that the WM structures soon become isointense or hyperintense compared to GM in T1-weighted images, whereas the signal of that structure is still high in T2-weighted images. The WM becomes dark on T2-weighted images only in more advanced stages of myelination [35]. This data corroborate our findings, once that both suggest higher sensibility of T1 acquisition to detect earlier WM changes (increase or decrease of signal).

From the comparison between the right and left groups we observed that left MTLE group presented more intense atrophy in bilateral temporal lobes. Some published data had demonstrated a wider pattern of atrophy in patients with left MTLE [36, 37], in association with a poor control of seizures in these patients. This contrast could be linked to major excitotoxic damage in dominant hemisphere's cells. Other studies showed more pronounced structural changes in right-sided MTLE, explaining this finding by the existence of different neuronal networks and pathophysiologic changes in temporolimbic structures in both hemispheres [38].

As possible limitation, we need to mention that MRI can have technical artifacts that are more evident in the base of the skull, which may influence in the detection of lesions in these areas. The use of masks refers to the usage of templates, the standard brain at which all the others are normalized to make possible the comparisons, without considering the individual neuroanatomical variability. However, it is important to consider that these problems apply to all the images, in all groups, in which images were acquired and processed in the same way, including the controls. Therefore, these possible sources of errors did not interfere significantly in the comparison between T1- and T2-weighted images or left and right MTLE patients, discussed above.

Others studies are necessary to compare patients with well-controlled and refractory epilepsy. The direct VBM analysis with these groups was not performed here, but we hypothesize that different pattern of atrophy may occur. 

## 5. Conclusions

In MTLE, T1-weighted images show more widespread pattern of atrophy of WM than T2-weighted images. T2-weighted images can be used to detect atrophy of WM, with a considerable superposition of findings compared with T1-weighted images, but demonstrating a more restricted pattern of atrophy. Studies with larger number of patients will be necessary to elucidate if there are really differences of signal intensity as measured by VBM between these two acquisitions.

## Figures and Tables

**Table 1:** Sample composition: patients, clinical information.

	Left group	Right group
Age average	43.96 ± 12.66	40.67 ± 12.75
First seizure (years)	15.33 ± 10.32	12.18 ± 8.87
Male	57.57%	28.57%
Female	42.43%	71.43%
Type of seizure:		
Partial simple	48.48%	42.85%
Partial complex	69.69%	66.66%
GTCS	51.51%	53.58%
Persistence of seizures since 2008		
Yes	87.5%	80.0%
No	12.5%	20.0%

**Table 2:** Areas with WM atrophy in patients with left HA, from T1-weighted analysis.

Left atrophy T1				
Number clusters	*T*	*P* (unc)	*x*, *y*, *z*	Areas
101294	7.47	<0.0001	−20, −33, 12	Left and right frontal lobes, corpus callosum, left and right cerebellum, thalamus, left and right hippocampus, left and right parahippocampal gyrus, left and right caudate, left and right insula, medulla, left amigdale
	7.01	<0.0001	−26, −33, 2	
	6.47	<0.0001	−4, −11, 17	
68	3.69	<0.0001	−46, 42, −12	Right frontal inferior orbital gyrus, middle frontal gyrus, inferior frontal gyrus
146	3.60	<0.0001	−9, 51, 15	Right medial frontal gyrus, frontal superior medial, cingulum anterior, superior frontal gyrus.
58	3.51	<0.0001	8, 46, 21	Left medial frontal gyrus, frontal medial superior gyrus

**Table 3:** Areas with WM atrophy on patients with left HA, from T2-weighted analysis.

Left atrophy T2				
Number clusters	*T*	*P* (unc)	*x*, *y*, *z* (mm)	Areas
	7.32	0.000	−22, −26, −16	Left and right parahippocampal gyrus, thalamus, cingulate gyrus, corpus callosum, left and right hippocampus, insula, caudate
51784	6.39	0.000	−33, −0, −31	
	6.24	0.000	−2, −12, 12	
	5.77	0.000	9, −39, −64	Left and right cerebellum
5105	5.00	0.000	15, −57, −66	
	4.87	0.000	−2, −50, −58	
318	3.87	0.000	66, −30, 9	Left superior temporal and medial gyrus
	3.67	0.000	58, −38, 9	
72	3.85	0.000	9, −2, 75	Right superior frontal gyrus
84	3.84	0.000	26, 1, 71	Right superior and middle frontal gyrus
58	3.71	0.000	32, −75, 50	Right parietal inferior and superior area
646	3.65	0.000	15, −54, −33	Right cerebellum
70	3.62	0.000	−14, −65, −10	Left lingual gyrus, left occipital lobe, and right cerebellum anterior lobe
224	3.58	0.000	−33, −68, −61	Left cerebellum
205	3.54	0.000	−26, −80, −4	Left occipital lobe, left fusiform, and lingual gyrus
56	3.50	0.000	−42, −2, 63	Left middle frontal gyrus, left frontal lobe
78	3.29	0.001	12, 4, −6	Right pallidum, right lentiform nucleus, right putamen, and right caudate

**Table 4:** Areas with WM atrophy on patients with right HA, from T1-weighted analysis.

Right atrophy T1				
Number clusters	*T*	*P* (unc)	*x*, *y*, *z*{mm}	Areas
	8.22	0.000	18, −35, 12	Left and right frontal lobes, corpus callosum, thalamus, right and left cerebellum, right and left cingulum gyrus, right and left hippocampus and parahippocampal gyrus, precentral gyrus, right and left insula, right caudate, medulla right and left amygdala
130229	8.13	0.000	32, −38, −6	
	7.77	0.000	24, −36, −1	
1434	4.50	0.000	6, −39, −67	Right cerebellum
	3.67	0.000	−8, −51, −69	
60	3.87	0.000	−18, −80, 36	Left parietal lobe, left cuneus, left occipital superior
72	3.54	0.000	−14, −84, 16	Left occipital lobe, cuneus, calcarine area
61	3.49	0.000	18, −74, 45	Right parietal lobe

**Table 5:** Areas with WM atrophy in patients with right HA, from T2-weighted analysis.

Right atrophy T2				
Number clusters	*T*	*P*	*x*, *y*, *z*	Areas
	7.92	0.000	28, −35, −13	Right frontal and temporal lobes, corpus callosum, cingulate gyrus, precentral gyrus, right and left parahippocampal gyrus, thalamus, right and left hippocampus, right insula, right fusiform, caudate, right and left precuneus, right amygdala
57887	7.34	0.000	22, −29, −10	
	6.60	0.000	14, −35, 2	
351	4.11	0.000	−45, −51, −55	Left cerebellum
	3.85	0.000	−3, −81, 11	Left occipital lobes
559	3.65	0.000	−15, −77, 24	
	3.53	0.000	−27, −80, 21	
59	3.74	0.000	−42, −35, 64	Left parietal lobe, postcentral gyrus
133	3.72	0.000	46, −56, −54	Right cerebellum
317	3.70	0.000	−46, −5, −22	Left temporal lobe, left middle and inferior temporal gyrus, left hippocampus
224	3.58	0.000	18, −56, −64	Undefined
	3.36	0.000	18, −69, −63	
75	3.40	0.000	−34, 21, −36	Superior temporal gyrus
95	3.39	0.000	−38, −69, 5	Left occipital lobe, middle occipital gyrus
	3.27	0.001	−33, −69, −3	
56	3.33	0.001	−16, −83, −3	Left calcarine, right lingual
